# Beyond mere novelty: Categorization drives differentiation

**DOI:** 10.1111/bjso.70120

**Published:** 2026-08-03

**Authors:** Patrick Rothermund, Roland Deutsch

**Affiliations:** ^1^ Julius‐Maximilians‐Universität Würzburg Würzburg Germany

**Keywords:** category learning, differentiation, distinctiveness, novelty, stereotypes

## Abstract

Stereotype formation is shaped by *differentiation*, that is, the tendency to prioritize attributes that render a novel group distinct from comparison groups. While differentiation is well documented, it remains unclear whether it is intrinsic to categorization or whether it is a byproduct of a general processing advantage of novel stimuli. We conducted three preregistered experiments to disentangle these accounts. In all experiments, participants learned a set of social traits that were either assigned to a single group or distributed across two groups. Crucially, novel traits presented in the second half of the stimulus set marked intergroup distinctiveness only in the two‐groups condition while being equally novel in the single‐group condition. In line with a category‐based account of differentiation, recall for novel traits was enhanced in the two‐groups condition compared to the single‐group condition (Exp. 1 & 3) and an additional control condition without groups (Exp. 3). Moreover, novel traits were judged as more typical for their group than initially learned traits, but only in the two‐groups condition (Exp. 2). These findings suggest differentiation is not a fixed response to novelty but a genuine categorization effect, serving a functional role in distinguishing groups. We discuss implications for category learning and stereotype formation.

**Key Points**

**What is already known?**

- Differentiation is a key process in stereotype formation: Distinctive attributes are prioritized when forming impressions of novel groups.
- As a result, real‐world novel groups (e.g. outgroups or minorities) may become associated more strongly with their distinctive attributes relative to the attributes they share with comparison groups (e.g. ingroups or majorities).

**What does this study add?**

- This study identifies the cognitive origin of differentiation by contrasting a novelty‐driven account with a categorization‐driven account. Across three experiments, the findings support the latter, suggesting that distinctive attributes are prioritized because they are functional for categorization rather than due to their mere novelty.
- The findings imply that uncommon behaviours may be especially memorable, and more likely to become associated with a group, when displayed by outgroup or minority members than when the same behaviours are displayed by ingroup or majority members.

Stereotypes are a fundamental part of human cognition, enabling individuals to simplify complex social environments by attributing certain traits to social groups. However, this comes at a cost, as stereotypes can be rigid and inaccurate (Bodenhausen et al., 2012). Understanding how such representations form is therefore central to social psychological research. One key process in stereotype formation is *differentiation*, which refers to the prioritization of distinctive attributes when learning information about novel groups (Alves et al., 2018; Rothermund & Deutsch, 2025; Woitzel & Alves, 2024). In this article, we contrast two accounts of differentiation. The first account views differentiation as a byproduct of a stimulus‐driven bias towards novelty, whereby novel information receives more attention and is better remembered. The second account views differentiation as a process specific to category learning, whereby differentiating traits are prioritized due to their functional role in setting a novel group apart from known groups. Anticipating our results, three preregistered experiments support the latter account and offer important implications for theories of category learning and stereotype formation.

## DIFFERENTIATION IN CATEGORY LEARNING

When describing social groups, individuals often emphasize characteristics that distinguish one group from another (Eyal & Epley, 2017; Ford & Stangor, 1992; Rothermund & Deutsch, 2025; Spears, 2002). For example, women may be described as warm and men as dominant, as these attributes are perceived to differentiate gender categories. By contrast, neither women nor men are likely described as having two legs, an attribute that is prevalent across gender categories but not distinctive for either. This emphasis on intergroup distinctiveness has multiple causes. For instance, it may serve the identity‐related motive to render ingroups positively distinct from outgroups (Tajfel & Turner, 1986). Additionally, it may support system‐justifying beliefs that ascribe distinct roles to social groups (Jost et al., 2004). Moreover, it may reflect adherence to conversational norms that prescribe the communication of diagnostic information (Grice, 1975).

The maximization of intergroup distinctiveness also emerges in the absence of higher‐order social or communicative forces. A large body of research shows that observers systematically overweight distinctive information when forming representations of novel concepts, categories or social groups, for which identity, justice or communicative concerns are not relevant (Alves et al., 2018; Houston et al., 1989; Houston & Sherman, 1995; Krueger & Rothbart, 1990; Rothermund & Deutsch, 2024, 2025; Sherman et al., 2009; Woitzel & Alves, 2024). These studies use learning paradigms in which participants acquire information about two groups in sequential order. The central finding is that traits distinguishing the second‐learned group are prioritized more strongly than equally distinctive traits of the first‐learned group. This asymmetry indicates that biases towards distinctiveness arise during learning: Only for the second‐learned group are distinctive traits identifiable as such at the time of encoding because a relevant comparison category is already available. For instance, in a study by Alves et al. (2018), participants learned attributes of two fictitious groups in sequential order. When the second‐learned group shared negative attributes with the first‐learned group but had unique positive attributes, it was preferred over the first‐learned group; but when it shared positive attributes but had unique negative attributes, the first‐learned group was preferred. Similar results were obtained in a study by Rothermund and Deutsch (2025), where participants evaluated statements that ascribed traits to two previously learned fictitious groups as true or false. For the second‐learned group, statements were more likely accepted as true when ascribing unique (vs. shared) attributes to the group. These results indicate that unique attributes of novel groups are prioritized during learning.

## TWO ACCOUNTS OF DIFFERENTIATION

While there is ample evidence that learning about a novel group is biased towards information that differentiates it from known comparison groups, the root cause of differentiation is yet unclear. In the present work, we distinguish two accounts of differentiation that have not yet been systematically tested against each other. One possibility is that differentiation is a purely stimulus‐driven process, reflecting a fundamental cognitive bias: novel stimuli stand out from ongoing streams of information and receive prioritized processing, as shown across several experimental paradigms. In *novel popout* studies, participants briefly view four‐word arrays. When one word is novel and the others are repeated, localization is more accurate for the novel word, indicating an attentional bias towards new items (Johnston et al., 1990; McCarthy & Reed, 2024). *Repetition blindness* refers to the related phenomenon that briefly presented stimuli are more likely to be detected when they are novel rather than repeated (Kanwisher, 1987; Morris & Harris, 2004). Similarly, in *oddball paradigms*, rare stimuli evoke larger event‐related potentials (Picton, 1992) and are perceived to last longer (Tse et al., 2004) than common stimuli. Together, these findings suggest that novel information commands special processing, potentially resulting in better memory (cf. Cowan et al., 2024). While this bias does not require the presence of any category structure, it may explain intergroup differentiation: Unique attributes of novel groups may be prioritized not because they mark intergroup distinctiveness, but because they are rare in the broader information ecology. In other words, the novelty‐based account suggests that differentiation is not inherently specific to category learning but emerges as an epiphenomenon of a general processing advantage for novel information. Without formalizing such an account explicitly, previous work has linked differentiation to attentional biases towards novel or unexpected information (Huang & Sherman, 2018).

The other possibility is that the prioritization of distinctive stimuli is not elicited by their mere novelty but requires knowledge of the categorical structure. From this perspective, differentiation reflects a functional aspect of category learning by establishing meaningful distinctions between categories. An early framework anticipating this idea is Self‐Categorization Theory (SCT; Turner et al., 1987), which conceptualizes categories as relational constructs that are defined by contrast to comparison categories. This notion is formalized in the meta‐contrast principle, according to which groups are identified to the extent that perceived differences between groups exceed perceived differences within groups. Although originally proposed as an account of how category boundaries are established, SCT also implies that category representations are structured around information that maximizes intergroup distinctiveness. Similar ideas have been echoed in other accounts of categorization and stereotype formation that emphasize contrast maximization as a means of rendering categories coherent and meaningful (Rosch, 1978; Spears, 2002). At a more general level, goal‐based theories of conceptual processing view category representations as adapting to the demands of the current situation (Barsalou, 2017; Barsalou et al., 2018). From this perspective, differentiation during learning may implement a current goal of rendering groups distinct from one another. At a more mechanistic level, cognitive research on category learning has documented *highlighting* (Kruschke, 2003), which refers to the phenomenon that cues with incremental value for discriminating categories acquire enhanced weight because they provide unique predictive information. Despite their different emphases, these theoretical approaches converge on the idea that distinctive information is prioritized because it serves a functional role within a categorical structure.

At the same time, these approaches were not developed to explain the asymmetric learning effects observed in contemporary differentiation paradigms. Specifically, SCT and goal‐based theories emphasize the flexibility of category representations and thus do not directly address learning biases such as the asymmetric perception of first‐ and second‐learned groups that are central to contemporary studies of differentiation (Alves et al., 2018; Rothermund & Deutsch, 2025; Woitzel & Alves, 2024). Conversely, highlighting provides a mechanistic account of how distinctive cues acquire enhanced weight but has been studied in paradigms involving active category prediction rather than passive impression formation (Medin & Edelson, 1988). Thus, while both perspectives are broadly consistent with the idea that category representations become organized around distinctive information, they do not by themselves establish whether differentiation arises because learners encode information in relation to category boundaries, or because distinctive attributes tend to be novel relative to previously encountered information. Notably, the theoretical narrative in differentiation research is also rather agnostic, describing differentiation as a fundamental cognitive principle but not addressing that distinctiveness is often confounded with novelty (Alves et al., 2018). Thus, it remains an open empirical question whether differentiation is inherently tied to categorization or could arise as an epiphenomenon of novelty‐driven processing.

## THE PRESENT STUDY

The present study contrasts two accounts of differentiation. The novelty‐based account proposes that distinctive traits of novel groups are prioritized because of their overall novelty within the broader information ecology. By contrast, the categorization‐based account proposes that distinctive traits are prioritized because they serve a functional role in distinguishing one group from another. Importantly, in the present work, novelty is operationalized as prior non‐occurrence within the learning sequence. This is consistent with common differentiation paradigms in which distinctive stimuli are operationalized by their uniqueness within the evolving categorization (e.g. Alves et al., 2018). Accordingly, novelty in the present case is determined by whether a trait has been encountered before, not by its semantic relation to other traits.^1^

**FIGURE 1:**
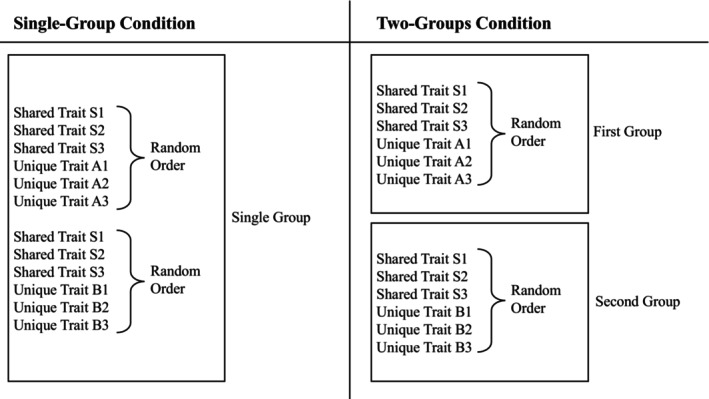
Illustration of the trait distribution in the learning set‐up employed in all experiments. The trait structure was identical across both conditions. In the two‐groups condition, the first and second set of six attributes marked different groups, while in the single‐group condition, both sets of six attributes belonged to the same group.

We conducted three preregistered experiments to test a key implication of the categorization‐based account: That enhanced processing of novel traits depends on their role in rendering a novel group distinct from a comparison group. In all experiments, we used a modified version of the trait‐learning paradigm introduced by Alves et al. (2018). Participants were sequentially presented with twelve members of either one or two fictitious social groups, with each member being described by a single trait. In the two‐groups condition, each group was characterized by three unique traits and three traits shared with the other group. In the single‐group condition, all twelve traits belonged to the same group. However, the trait structure was matched across conditions: the first and second sets of six exemplars each included three unique traits and three shared traits (see Figure 1). Thus, unique traits in the second set were always novel, but only in the two‐groups condition did they distinguish a new group from a known group. This learning set‐up allowed to disentangle genuine categorization effects from mere novelty effects. The novelty‐based account predicts that novel traits should receive enhanced processing regardless of condition. By contrast, the categorization‐based account predicts enhanced processing specifically when novel traits support intergroup differentiation, as is the case for unique traits of the second group in the two‐groups condition, but not for the single‐group condition.

We tested two related predictions that follow uniquely from the categorization‐based account. The first prediction concerns memory. If novel information receives enhanced processing because it serves a differentiating function, novel traits should be better recalled when they belong to a new group than when they follow previously learned traits from the same group. Experiments 1 and 3 tested this prediction, with Experiment 3 including an additional control condition where the traits were presented without any reference to groups. The second prediction concerns group representations. If differentiation increases the representational weight of distinctive information, novel traits should be judged as more typical for their group than traits learned earlier, but only when they belong to a new group. Experiment 2 tested this prediction.

## EXPERIMENT 1

### Method

#### Participants

Following the preregistered protocol (https://aspredicted.org/2xvr‐jnvr.pdf), we implemented a sequential analysis plan (Lakens, 2014) involving a maximum of 160 participants. An interim analysis was conducted after collecting data from 80 participants. As preregistered, we stopped data collection after the interim analysis as the focal contrast test reached significance (see below). To correct for multiple testing, we applied a Pocock correction (Lakens, 2014), adjusting the alpha level to *α* = .030 for the interim analysis (and the final analysis, if it would have been conducted). Sample size was determined by heuristic judgement, with a sensitivity analysis revealing that the sequential analysis plan allowed us to detect an effect size of *d* = 0.19 with a power of *β* = .8. As preregistered, we excluded datasets from five participants who did not recall at least two traits correctly. Thus, the final sample size was *n* = 75 (31 women, 41 men, 3 non‐binary, M_age_ = 34.0, SD_age_ = 10.4). All participants were recruited via Prolific, resided in Germany and stated that their first language was German. Participants received £0.60 for completing the approximately four‐minute experiment.

#### Design

The experiment employed a 3 (trait type: shared vs. unique‐first vs. unique‐second) × 2 (group condition: single‐group vs. two‐groups) design. Trait type was a within‐subjects factor, while group condition was manipulated between‐subjects.

#### Procedure

Participants accessed the online experiment, gave informed consent and provided demographic information. They were then asked to imagine a fictitious scenario set in the year 2080, where they travelled to a distant planet inhabited by aliens. In the single‐group condition, participants were told the planet was home to one alien tribe and that they would encounter twelve aliens from this tribe, each represented by a personality trait. In the two‐groups condition, participants were told the planet was home to two tribes (‘Tribe X’ and ‘Tribe Y’) and that they would encounter six aliens from each tribe, each represented by a personality trait. Participants were instructed that their task was to form an accurate impression of the alien tribe(s). Next, participants viewed twelve slides, each showing a single trait. In the two‐groups condition, they were told at the start that the first six traits belonged to Tribe X, and after six slides, another slide indicated that the next six traits belonged to Tribe Y. Nine mildly positive traits (e.g. ‘friendly’) were used throughout the experiment (full list available on OSF). For each participant, traits were randomly assigned to three roles: three unique‐first, three unique‐second and three shared traits. The first six slides included the unique‐first and shared traits in random order; the second six included the unique‐second and shared traits, also in random order. This structure was identical across conditions. After the learning phase, participants completed a recall test (see *Dependent variable*), were thanked and redirected to Prolific.

#### Dependent variable

Trait recall was measured in a surprise memory test and served as the only dependent variable. Participants were asked to type all the traits they could remember into a textbox. For each participant, we determined the number of traits recalled from each category (unique‐first, unique‐second and shared traits).

### Results

**FIGURE 2:**
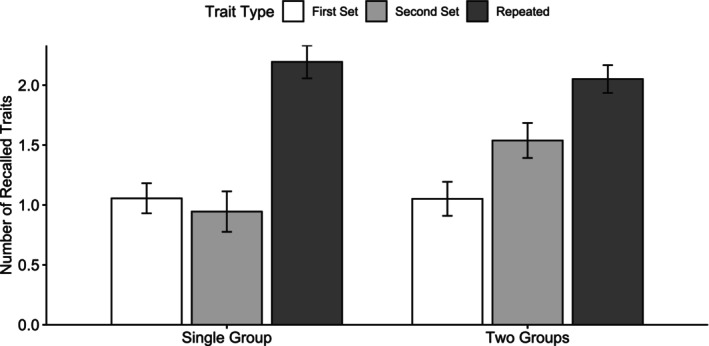
Mean number of recalled traits of each type as a function of group condition (Experiment 1). Error bars represent standard error of means.

All data and the analysis script of Experiment 1 are available on OSF. Descriptive results are shown in Figure 2. We analysed recall performance using a mixed analysis of variance (ANOVA) including the factors trait type and group condition. There was no significant main effect of group condition, *F*(1, 73) = 2.09, *p* = .153, η^2^_p_ = 0.03, indicating that the total number of recalled traits did not differ between conditions. However, a significant interaction emerged between both factors, *F*(2, 146) = 3.57, *p* = .031, η^2^_p_ = 0.05, indicating that group condition influenced recall differently depending on the type of trait. Specifically, and as predicted, contrast tests revealed that participants in the two‐groups condition recalled more unique traits from the second set than those in the single‐group condition, *t*(73) = 2.67, *p* = .005, *d* = 0.62 (one‐tailed).^2^ No differences between group conditions were observed for unique traits from the first set, *t*(73) = 0.02, *p* = .982, *d* = 0.01 (two‐tailed), or for shared traits, *t*(73) = 0.80, *p* = .425, *d* = 0.19 (two‐tailed).

### Discussion

The goal of Experiment 1 was to test whether the introduction of a second group enhances the processing of its unique traits beyond the mere novelty status of these traits. Consistent with this hypothesis, participants recalled more novel traits when they were associated with a second group than when they were associated with a previously known group. By contrast, recall for other traits did not differ between the single‐group and two‐groups conditions. This pattern strongly supports the categorization‐based account of differentiation over the novelty‐based account. Simply encountering novel information was not sufficient to boost recall; instead, novelty conferred a processing advantage only when it served a differentiating function within a categorical structure.

Beyond influencing overall memory, differentiation is of particular interest because it shapes stereotypes. To approximate this level of representation, Experiment 2 used the same learning paradigm but assessed participants' impressions of the groups instead of their recall performance. Specifically, participants rated the typicality of each trait for the groups, providing a more direct measure of how distinctive information contributes to emerging group representations.

## EXPERIMENT 2

### Method

#### Participants

We preregistered a sequential analysis plan similar the one used in Experiment 1 (https://aspredicted.org/gmjq‐fqmr.pdf). This time, the focal interaction test (see below) did not reach the adjusted significance level (*α* = .030) at the interim analysis conducted after 80 participants (*p* = .035). Thus, we collected data from another 80 participants, resulting in a final sample size of *n* = 160 (56 women, 102 men, 2 non‐binary, M_age_ = 33.3, SD_age_ = 9.3). Sample size was determined by heuristic judgement, with a sensitivity analysis revealing that the sequential analysis plan allowed us to detect an effect size of η^2^_p_ = 0.04 with a power of *β* = 0.8. All participants were recruited via Prolific, resided in Germany and stated that their first language was German. Participants received £0.60 for completing the approximately four‐minute experiment.

#### Design

The experiment employed a 2 (trait type: unique‐first vs. unique‐second) × 2 (group condition: single‐group vs. two‐groups) design. Trait type was a within‐subjects factor, while group condition was manipulated between‐subjects. As in Experiment 1, shared traits were included in the learning set; however, ratings for these traits were analysed only exploratorily (see below). The reason is that they did not permit a parallel comparison across conditions: In the single‐group condition, shared traits were rated once, whereas in the two‐groups condition, they were rated separately for each group.

#### Procedure

The procedure was identical to Experiment 1, with the only difference that participants made typicality judgements instead of doing a recall test (see *Dependent variable*).

#### Dependent variable

After the learning phase, participants rated the typicality of the learned traits for the respective group(s) on a continuous scale (1–101). In the single‐group condition, participants rated the typicality of nine traits (three unique‐first, three unique‐second, three shared) presented in random order. In the two‐groups condition, they rated the typicality of six traits per group (three unique and three shared each), with both group order and trait order randomized. Ratings of shared traits were only used for exploratory analysis.

### Results

**FIGURE 3:**
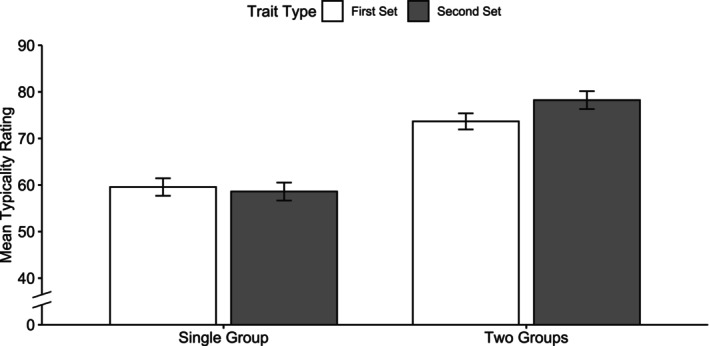
Mean typicality ratings for traits uniquely presented in the first and second set as a function of group condition (Experiment 2). Error bars represent standard error of means.

All data and the analysis script of Experiment 2 are available on OSF. Descriptive results are shown in Figure 3. We analysed typicality ratings using a mixed ANOVA including the factors trait type and group condition. Unique traits in the two‐groups condition were judged as more typical for their groups compared to unique traits in the single‐group condition, as indicated by a main effect of group condition, *F*(1, 158) = 46.99, *p* < .001, η^2^_p_ = 0.23. Confirming our main prediction, a significant interaction emerged between both factors, *F*(1, 158) = 8.39, *p* = .004, η^2^_p_ = 0.05. Specifically, contrast tests revealed that in the two‐groups condition, unique traits of the second set were judged as more typical for their group than unique traits of the first set, *t*(158) = 3.40, *p* < .001, *d*_*z*_ = 0.27 (one‐tailed). By contrast, no such difference was observed in the single‐group condition, *t*(158) = 0.71, *p* = .479, *d*_*z*_ = 0.06 (two‐tailed). For exploratory purposes, we also examined shared traits in the two‐groups condition (as described, the single‐group condition does not provide a parallel comparison, as shared traits were rated only once in this condition). Shared traits were judged equally typical for the first and second group (M_*first*_ = 72.7, M_*second*_ = 73.3), *t*(80) = 0.37, *p* = .715, *d*_*z*_ = 0.04 (two‐tailed).

### Discussion

The goal of Experiment 2 was to test whether novel traits are prioritized in group representations only when they serve to distinguish the group from a known comparison group. Supporting this hypothesis, participants rated novel traits in a second‐learned group more typical than unique traits of a previously learned group. By contrast, novel traits were not seen as more typical when both trait types belonged to the same group. These findings add to Experiment 1 by showing that the processing advantage of differentiating traits extends beyond recall to typicality judgements, which reflect stereotype formation more directly. Again, the results are only consistent with a categorization‐based account of differentiation, but not with a purely novelty‐based account that would predict a prioritization of novel traits regardless of their role in intergroup differentiation. Finally, exploratory analyses revealed that shared traits are not augmented in representations of a second group compared to a first group, further underlining that stereotype formation follows differentiation.

Experiments 1 and 2 provide converging evidence that differentiation reflects more than a general processing advantage of novel information, yet one alternative interpretation deserves consideration. Specifically, the single‐group condition may encourage participants to maintain a coherent impression of a single social category, thereby reducing the weight assigned to newly introduced information. If so, the differences observed in Experiments 1 and 2 could reflect attenuated processing of novel traits in the single‐group condition rather than enhanced processing in the two‐groups condition.^3^ Experiment 3 was designed to adjudicate between these possibilities by introducing a no‐groups control condition. In this condition, participants were presented with the same sequence of traits but received no instructions referring to any groups. Instead, they were simply instructed to attend to each trait (cf. Schul, 1983).

As in Experiment 1, we measured recall performance. If the single‐group condition suppresses the processing of novel information, recall performance in the no‐groups condition should resemble that in the two‐groups condition. In contrast, if the enhanced recall observed previously reflects categorization‐based differentiation, performance in the no‐groups condition should resemble that in the single‐group condition and remain below that observed in the two‐groups condition.

## EXPERIMENT 3

### Method

#### Participants

Following the preregistered protocol, we collected data from 240 participants (https://aspredicted.org/vm5tw4.pdf).^4^ This sample size matched the power of Experiment 1, allowing us to detect an effect size of *d* = 0.19 with a power of *β* = 0.8. As preregistered, we excluded datasets from eight participants who did not recall at least two traits correctly. Thus, the final sample size was *n* = 232 (60 women, 170 men, 2 non‐binary, M_age_ = 30.0, SD_age_ = 9.7). All participants were recruited via Prolific, resided in Germany and stated that their first language was German. Participants received £0.70 for completing the approximately four‐minute experiment.

#### Design

The experiment employed a 3 (trait type: shared vs. unique‐first vs. unique‐second) × 3 (group condition: single‐group vs. two‐groups vs. no‐groups) design. Trait type was a within‐subjects factor, while group condition was manipulated between‐subjects.

#### Procedure

The procedure was identical to Experiment 1, except for the inclusion of the no‐groups condition. In this condition, any references to alien tribe(s) were omitted. Instead, participants were instructed that they were going to read twelve personality traits and that their task was to attend to each trait carefully.

#### Dependent variable

The dependent variable was identical to Experiment 1.

### Results

**FIGURE 4:**
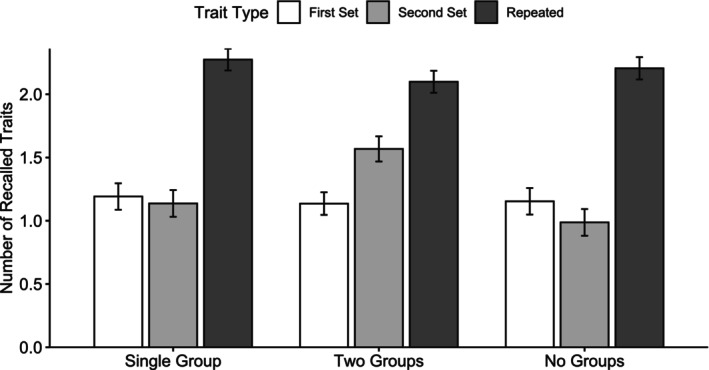
Mean number of recalled traits of each type as a function of group condition (Experiment 3). Error bars represent standard error of means.

All data and the analysis script of Experiment 3 are available on OSF. Descriptive results are shown in Figure 4. As in Experiment 1, we analysed recall performance using a mixed ANOVA including the factors trait type and group condition. There was no significant main effect of group condition, *F*(2, 229) = 1.49, *p* = .228, η^2^_p_ = 0.01, indicating that the total number of recalled traits did not differ between conditions. However, a significant interaction emerged between both factors, *F*(4, 458) = 5.21, *p* < .001, η^2^_p_ = 0.04, indicating that group condition influenced recall differently depending on the type of trait. Specifically, and as predicted, contrast tests revealed that participants in the two‐groups condition recalled more unique traits from the second set than those in the single‐group condition, *t*(229) = 2.94, *p* = .002, *d* = 0.47 (one‐tailed), and more than those in the no‐groups condition, *t*(229) = 4.02, *p* < .001, *d* = 0.65 (one‐tailed), with no differences between the single‐group condition and the no‐groups condition, *t*(229) = 1.01, *p =* .313, *d* = 0.16 (two‐tailed). No differences between group conditions were observed for unique traits from the first set (all *p*s ≥ .692, two‐tailed) or for shared traits (all *p*s ≥ .158, two‐tailed).

### Discussion

The goal of Experiment 3 was to rule out an alternative explanation for the results of Experiments 1 and 2. Specifically, it was possible that the single‐group condition attenuated the processing of novel traits, supporting a coherent impression of a single group. To test this possibility, Experiment 3 included a no‐groups condition in which participants encoded the same trait information without engaging in impression formation. Recall of novel traits in this condition was similar to the single‐group condition and below the two‐groups condition. Thus, the memory advantage for novel traits in the two‐groups condition cannot be attributed to reduced processing in the single‐group condition. Instead, the results further support the conclusion that novelty enhances processing only when it serves to differentiate one group from another.

## GENERAL DISCUSSION

Stereotypes of novel groups tend to form around distinctive attributes. Across three experiments, we provide evidence that this differentiation process is not due to a general bias towards novel information but is selectively triggered by situations that allow distinguishing a new group from an existing one. Specifically, novel traits are better remembered when they are linked to a second group than when they are linked either to a familiar group or to no group at all. Moreover, novel traits are rated more typical for their group than previously learned traits, but only when they are linked to a second group. Together, this suggests that differentiation crucially depends on the presence of a meaningful category structure.

The present findings have concrete implications for the formation of stereotype content. Specifically, they suggest that not all rare or novel attributes are equally influential across groups. Instead, they might play a particularly strong role in shaping stereotypes of novel groups, where such features contribute to differentiating the group from familiar comparison groups. Prior research has argued that outgroups and minority groups typically qualify as novel groups that are perceived in comparison to ingroups and majority groups. In contrast, stereotypes of ingroups and majority groups are often formed in relative isolation (Alves et al., 2018; Bergh & Lindskog, 2019). As a result, uncommon traits observed in outgroups and minority groups are more likely to be overweighted in corresponding stereotypes. In contrast, when uncommon traits are observed in ingroups and majority groups, they do not affect stereotypes to the same degree, contrary to what would be expected from a purely novelty‐based process. Thus, polarized perceptions of outgroups and minorities might be even more prevalent than previously recognized. Moreover, because rare attributes are often negative, this corroborates a consistent evaluative disadvantage for outgroups and minorities (Alves et al., 2018; Unkelbach et al., 2020).

The present study makes a theoretical contribution by clearly distinguishing novelty‐based biases from genuine categorization effects, a distinction that has not been coherently addressed in previous literature. Some work has taken an agnostic stance, characterizing differentiation as a hard‐wired cognitive principle without addressing that the distinctiveness of social traits is often confounded with their novelty (Alves et al., 2018). Earlier theories proposed that differentiation arises as a direct consequence of categorization but did not consider stimulus novelty as a plausible alternative explanation (Turner et al., 1987). By contrast, other lines of research have linked intergroup differentiation to attentional biases towards novel or unexpected information (Huang & Sherman, 2018). The present work clarifies this mixed picture by demonstrating that categorization elicits differentiation effects that novelty biases alone cannot explain.

At the same time, the precise mechanisms by which categorization gives rise to differentiation remain to be specified. The present findings are compatible with accounts at different levels of analysis that converge on the idea that category representations become organized around functionally informative distinctions. At a mechanistic level, the findings are broadly consistent with the highlighting effect (Kruschke, 2003), extending this logic from active category prediction to passive impression formation. More generally, and consistent with goal‐based theories of conceptual processing (Barsalou, 2017; Barsalou et al., 2018), knowledge of a categorization might engage top‐down processes that bias learning towards maximizing intergroup distinctiveness. From this perspective, differentiation might be construed as an active effort. For example, social perceivers may expect salient comparison groups to differ in meaningful ways (Kramer et al., 2021) and may satisfy this expectation by overweighting traits that distinguish a novel group from known groups. Relatedly, perceivers may be epistemically motivated to differentiate comparison groups in order to render the categorization coherent or informative (Spears, 2002). Future research should test these possibilities more directly by manipulating such top‐down expectations and motivations. For instance, if a novel social group is expected to cooperate rather than compete with a known group, this may induce a goal to assimilate rather than differentiate the groups, thereby reducing differentiation and stereotype polarization.

Importantly, the present findings should not be taken as general evidence for the absence of novelty biases. As reviewed in the introduction, a large body of research demonstrates enhanced processing of novel information across diverse paradigms (e.g. Johnston et al., 1990; Kanwisher, 1987). Some features of the present paradigm may have attenuated such effects. First, novelty was operationalized via prior non‐occurrence but not semantic dissimilarity. In fact, the exclusively positive traits were rather semantically homogeneous. Second, the overall novelty structure of the learning sequence was such that critical traits competed with information that was itself relatively unfamiliar: All traits were initially novel, and even repeated traits had been encountered only once. In more naturalistic settings, novel information may therefore receive an additional processing advantage that was not captured here. However, this possibility strengthens rather than weakens the present conclusions. Even under conditions that may have reduced novelty‐based biases, we observed robust differentiation effects in the two‐groups condition. Thus, the present findings provide particularly strong evidence that intergroup differentiation cannot be reduced to novelty alone but depend on the presence of a contrastive category structure.

### Limitations

Despite the insights gained from the present research, two limitations should be noted. First, an important constraint is that the categorization manipulation (single group vs. two groups) is confounded with the amount of information provided per group (six vs. twelve attributes). Although this was a critical design choice to keep the overall stimulus structure constant, it leaves room for alternative interpretations. Specifically, one might argue from a Bayesian perspective that belief updating could be stronger when new information pertains to a smaller set of traits (i.e. one out of six rather than one out of twelve), potentially leading to an overweighting of information about the novel group. Relatedly, dividing the same information into two groups may have yielded a more chunked memory representation, thereby facilitating encoding or retrieval (Gobet et al., 2001). However, both accounts would predict enhanced learning for both shared and distinctive traits of the second group. This pattern is not observed. Instead, shared traits are not prioritized in the two‐groups condition relative to the one‐group condition, indicating that the selective advantage for distinctive traits in the two‐groups condition reflects differentiation elicited by categorization rather than a general increase in belief updating or memory organization. Nevertheless, future research may identify ways to disentangle categorization effects from novelty effects while avoiding this design confound altogether.

Second, our studies used artificial social groups and a highly controlled trait‐learning paradigm, limiting ecological generalizability. In this paradigm, the groups were described exclusively with positive traits, which does not reflect the more complex mix of positive, negative and ambivalent traits typical of real‐world stereotypes. While prior research suggests that differentiation operates robustly across trait valences (Alves et al., 2018) and may even underlie some apparent valence effects (Unkelbach et al., 2020), we cannot fully rule out the possibility that trait valence somehow moderates the differentiation process. Moreover, although the strictly separated learning sequence employed in the present paradigm was essential for isolating differentiation as a learning mechanism, real‐world social learning might often involve more interleaved exposure to different groups.

## CONCLUSION

The present research supports the idea that differentiation serves a functional role in category learning rather than being a mere byproduct of a novelty bias. This suggests that rare traits are disproportionately weighted in impressions of novel social groups, as these traits help distinguish the group from familiar comparison groups. Differentiation contributes to the formation of polarized and evaluatively skewed stereotypes, particularly for outgroups and minorities. Thus, further examining the psychological mechanisms underlying differentiation is key to understanding the formation and persistence of social bias.

## AUTHOR CONTRIBUTIONS

**Patrick Rothermund:** Writing – original draft; writing – review and editing; conceptualization; formal analysis; data curation. **Roland Deutsch:** Writing – review and editing; conceptualization.

## CONFLICT OF INTEREST STATEMENT

No conflict of interest.

## ETHICS STATEMENT

Informed consent was obtained from all participants included in the study. All procedures were performed in accordance with the ethical standards of the institution's Human Research Ethics Committee. A formal approval of the Ethics Committee is available.

## Data Availability

We report how we determined our sample sizes, all data exclusions, all manipulations and all measures in the study. Data, analysis scripts and materials of all experiments are available for download from the Open Science Framework: https://osf.io/35479/?view_only=4ee908f6db7046d0adedbf2858b1be66.

## Footnotes

^1^
An alternative conceptualization of novelty includes semantic distance, such that, for example the word ‘murder’, but not the word ‘honest’ is novel when following repeated presentations of the words ‘friendly’. However, this issue is orthogonal to the present distinction between the novelty‐based and categorization‐based account. If semantic similarity renders traits less novel, it also renders them less distinctive. Any attenuation arising from semantic similarity would therefore affect both mechanisms rather than selectively weakening one of them.

^2^
Consistent with the preregistered protocols of all experiments, focal contrasts corresponding to directional predictions derived from the categorization‐based account were tested one‐tailed, whereas contrasts not predicted by the account were tested two‐tailed. Applying either one‐tailed or two‐tailed tests throughout would not change the pattern of results, that is, all one‐tailed tests would remain significant when tested two‐tailed, and none of the two‐tailed tests would become significant when tested one‐tailed.

^3^
We are grateful to an anonymous reviewer for suggesting this alternative explanation and for proposing the outline of the experimental design used to test it.

^4^
We deviated from the sequential analysis plans used in Experiments 1 and 2 because Experiment 3 involved two focal tests. This made a standard sequential stopping rule based on repeated significance testing less straightforward, as interim analyses could have yielded significance for one focal test but not the other.
